# Time to positivity of blood culture is a risk factor for clinical outcomes in Staphylococcus aureus bacteremia children: a retrospective study

**DOI:** 10.1186/s12879-019-3993-4

**Published:** 2019-05-17

**Authors:** Yuanyuan Li, Qinyuan Li, Guangli Zhang, Huan Ma, Yi Wu, Qian Yi, Lili Jiang, Jiao Wan, Fengtao Suo, Zhengxiu Luo

**Affiliations:** 1 Key Laboratory of Pediatrics in Chongqing, Chongqing, China; 2 Department of Children’s Hospital of Chongqing Medical University of Education, Ministry of Education Key Laboratory of Child Development and Disorders, China International Science and Technology Cooperation base of Child development and Critical Disorders, Chongqing, China; 3 Department of Respiratory Medicine, Children’s Hospital of Chongqing Medical University, Chongqing, 401122 China

**Keywords:** Time to positivity, *S.aureus* bacteremia, Children

## Abstract

**Background:**

*Staphylococcus aureus* (*S. aureus*) is a common cause of bacteremia, which leads to significant morbidity and mortality. We investigated the relationship between time to positivity (TTP) and clinical outcomes in children with *S.aureus* bacteremia in the China.

**Methods:**

A retrospective study of *Staphylococcus aureus* bacteremia inpatient was performed in Children’s Hospital of Chongqing Medical University in China between 29 January 2014 and 29 August 2017. TTP and clinical parameters were determined and analyzed. The receiver operating characteristic (ROC) curves were plotted for optimal cut-off selection, multivariate logistic regression tests were performed to evaluate the association between TTP and clinical outcomes.

**Results:**

Overall, 84 cases were enrolled. We stated that in-hospital mortality is significantly higher in the early TTP (≤17 h) than in the late TTP (> 17 h) group (57.14% vs 7.14%, *P* = 0.000). Septic shock occurred in 57.14% of patients with early TTP and in 18.57% of patients with late TTP (*P* = 0.002). Detailed multivariate and statistical analysis revealed that early TTP, need for vasoactive agent were independent risk factors of in-hospital mortality; early TTP, need for vasoactive agent and APACHE II score ≥ 15 were independent risk factors of septic shock incidence in *S. aureus* bacteremia children.

**Conclusions:**

Overall, TTP of ≤17 h appeared to correlate with the worse outcomes for *S. aureus* bacteremia children. These results have important implications in the assessments and management of pediatric *S. aureus* bacteremia in a clinical setting.

**Trial registration:**

Retrospectively registered.

## Background

*Staphylococcus aureus* (*S. aureus*) is a common cause of bloodstream infections [1] and *S. aureus* bacteremia (SAB) leads to significant morbidity and mortality, both in adults and children [2–4]. The prevalence of SAB in the pediatric populations of industrialized nations is high, ranging from 8.4 to 30/100,000 person-years [5, 6]. Previous studies emphasized the importance of early laboratory detection of SAB [7, 8], and demonstrated the time to positivity (TTP) of blood culture may serve as a predictor of clinical outcomes for adult SAB patients [9–12]. TTP has also been studied in children’s bacteremia caused by *H. influenza* [13] *, S. pneumonia* [13, 14], and *N. meningitides* [15]. Several studies indicated that early TTP was a poor prognostic factor in adult SAB patients [10, 16, 17]. However, few studies have assessed the value of TTP in SAB children, the relationship between TTP and the clinical outcomes of SAB children remains unclear. Here we aim to evaluate the relationship between TTP and clinical presentations and outcomes, to explore the risk factors of in-hospital mortality, septic shock incidence in children with SAB.

## Methods

### Study design and patients

This study was performed in Children’s Hospital of Chongqing Medical University, a 1500-bed tertiary level III teaching hospital in Chongqing, China. SAB children hospitalized in Children’s Hospital of Chongqing Medical University between 29 January 2014 and 29 August 2017 were enrolled retrospectively. The inclusions had the following characteristics: (i) inpatients (not include the emergency department); (ii) age < 18 years; (iii) with ≥1 *S. aureus* blood culture positive; (iv) with systemic inflammation reaction syndrome status. The exclusion criteria included any of the following: (i) patients who were lost to follow-up; (ii) patients with incomplete clinical information; (iii) patients without TTPs in medical records.

### Definitions

*Staphylococcus aureus* bacteremia (SAB) was defined as at least 1 positive *S. aureus* blood culture with systemic inflammation reaction syndrome status (SIRS), SIRS was defined according to the criteria previous published on *Pediatr Crit Care Med* [18]. Time to positivity (TTP) was defined as the time period between blood incubation and the positive signal, whereby all individuals were included only once. When multiple cultures were positive, the shortest TTP was enrolled. Prior antibiotic exposure was considered when the antibiotic was used before hospital-admission, regardless of administration time and dosage, appropriateness was judged by latter susceptibility results. Immunosuppressants consisted of corticosteroid and other cytotoxic agents, it was considered when duration of use ≥1 month. Neutropenia in this manuscript was associated with chemotherapy, defined by ANC (absolute neutrophil count) < 6.0 × 10^9^/L (infants), < 1.0 × 10^9^/L (< 1 year old), and < 1.5 × 10^9^/L (≥1 year old). The source of infection consisted of pneumonia, bone and joint, skin or soft tissue, endocarditis, CNS (central nervous system) infection, incisions, catheter related infection (urethral catheters and thoracic drain tube), and multiple sites. Nosocomial infection was defined as *S. aureus* infection that occurred > 48 h after hospital (not include the emergency department) admission or < 48 h after discharge. The length of hospitalization was defined as the time period between admission and discharge. The classification of septic shock was defined according to the criteria previously published in JAMA [19].

### Microbiologic method

Approximately 3 mL of blood was inoculated into BACTEC plus aerobic bottles, which were then transported to the laboratory and immediately incubated in an automated continuous monitoring system. The BD diagnostic system was used for blood culture, which monitors CO_2_ production every 5 min by means of a fluorescent signal. Bottles with positive results were examined by Gram staining, and their contents were subcultured. Species identification and susceptibility tests were performed using Vitek identification and susceptibility cards (bioMe’rieux Vitek).

### Data collection

The collected data included TTP of blood culture, demographic characteristics (age, gender, weight), underlying conditions (hematologic malignancy, congenital heart disease, neutropenia, primary immunodeficiency, chronic kidney disease), hypoalbuminemia, APACHE (acute physiology and chronic health evaluation) II scores, sources of infection as mentioned earlier, prior antibiotic exposure, appropriate antibiotic exposure, immunosuppressants use, nosocomial infection, methicillin-resistance, need for invasive mechanic ventilation, need for vasoactive agent, length of hospitalization, the incidence of septic shock and in-hospital mortality.

### Clinical outcomes

The primary outcome was in-hospital mortality. Since previous studies suggested that septic shock meant worse outcomes in adult SAB patients [20], it was chosen as the second outcome.

### Statistical methods

The continuous variables were presented as medians, and the first and third quartiles; the Student’s t test, Chi-square test, or the Mann-Whitney U test were used for analysis. The categorical variables were presented as rates and incidences, and assessed by means of the *Χ*^2^ test or Fisher’s exact test. The log-rank test for Kaplan-Meier time-to-positivity analysis was constructed. Receiver operating characteristic (ROC) curves were plotted for the rate of sensitivity and 1-specificity. Youden’s Index was used for optimal cut-off selection, and the area under the curve (AUC) was calculated. The non-parametric test was constructed for the comparison of two groups. Univariate and multivariate logistic regression tests were constructed, for exploring the independent risk factors of septic shock and in-hospital mortality. Univariate logistic regression test was constructed for all independent risk factor exploration of in-hospital mortality, septic shock. Multivariate logistic regression test was constructed for the variates that showed differences in Univariate logistic regression test. The odds ratio (OR) and their 95% confidence interval (CI) were calculated for the statistically significant variables. All the tests were performed using SPSS (Version 19.0.0 for Windows), and *p*-value < 0.05 was considered to be statistically significant.

## Results

### Study population

**Fig. 1:**
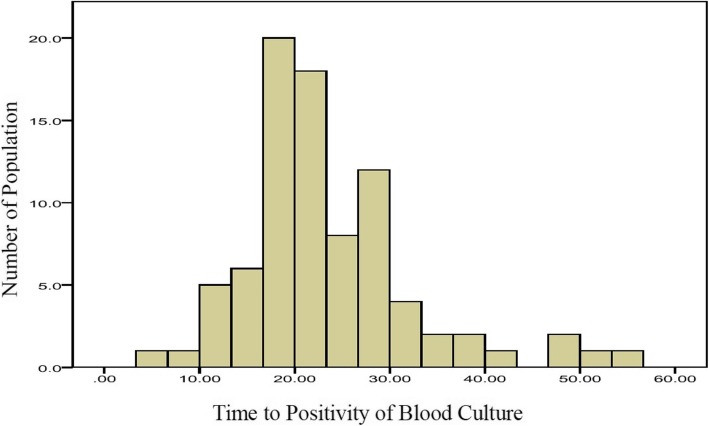
Bar chart of TTP in *Staphylococcus aureus* bacteremia children. Out of a total of 84 SAB children, the number of children in each TTP period was plotted against the corresponding TTP, as shown. Details of the quantification have been described in Methods

During the study period, 95 inpatient children with ≥1 *S. aureus* blood culture positive and systemic inflammation reaction syndrome status were enrolled retrospectively. Of them, 4 cases were lost to follow-up, 3 cases had incomplete clinical information and 4 cases missed their TTPs in medical records. Therefore, 84 cases were finally included in the study (Fig. 1).

### Clinical characteristics of ***S. aureus*** bacteremia in children

The median (IQR) age of the enrolled SAB children was 56.00 (10.95, 126.25) months, median (IQR) weight was 15.00 (8.00, 35.00) kilogram, and 65.5% (55/84) of them were male. Less than half of the patients (45.2%, 38/84) had underlying conditions. Among the 35 patients (41.7%) with prior antibiotic exposure, only 13 cases (15.5%) reported appropriate. Immunosuppressants were used in 20 cases (23.8%). The most frequent infection source was multiple sites (28.57%, 24/84), followed by pneumonia (27.38%), and bone or joint infection (15.48%). Methicillin-resistance was detected in 29 cases (34.5%), and nosocomial infection in 14 cases (16.7%).

### TTP of *S. aureus* bacteremia in children

**Fig. 2:**
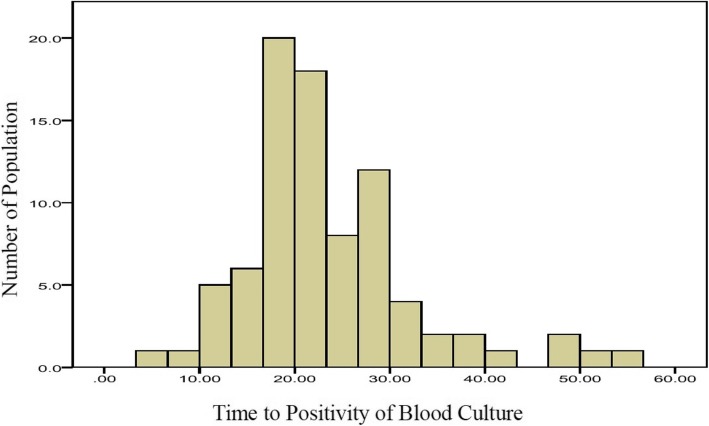
Flow diagram of the population. As shown, a total of 84 cases were enrolled according to the inclusion and exclusion criteria, and then divided into early detection group (TTP ≤ 17 h) and late detection group (TTP > 17 h). The clinical characteristics of each group were then determined

**Fig. 3:**
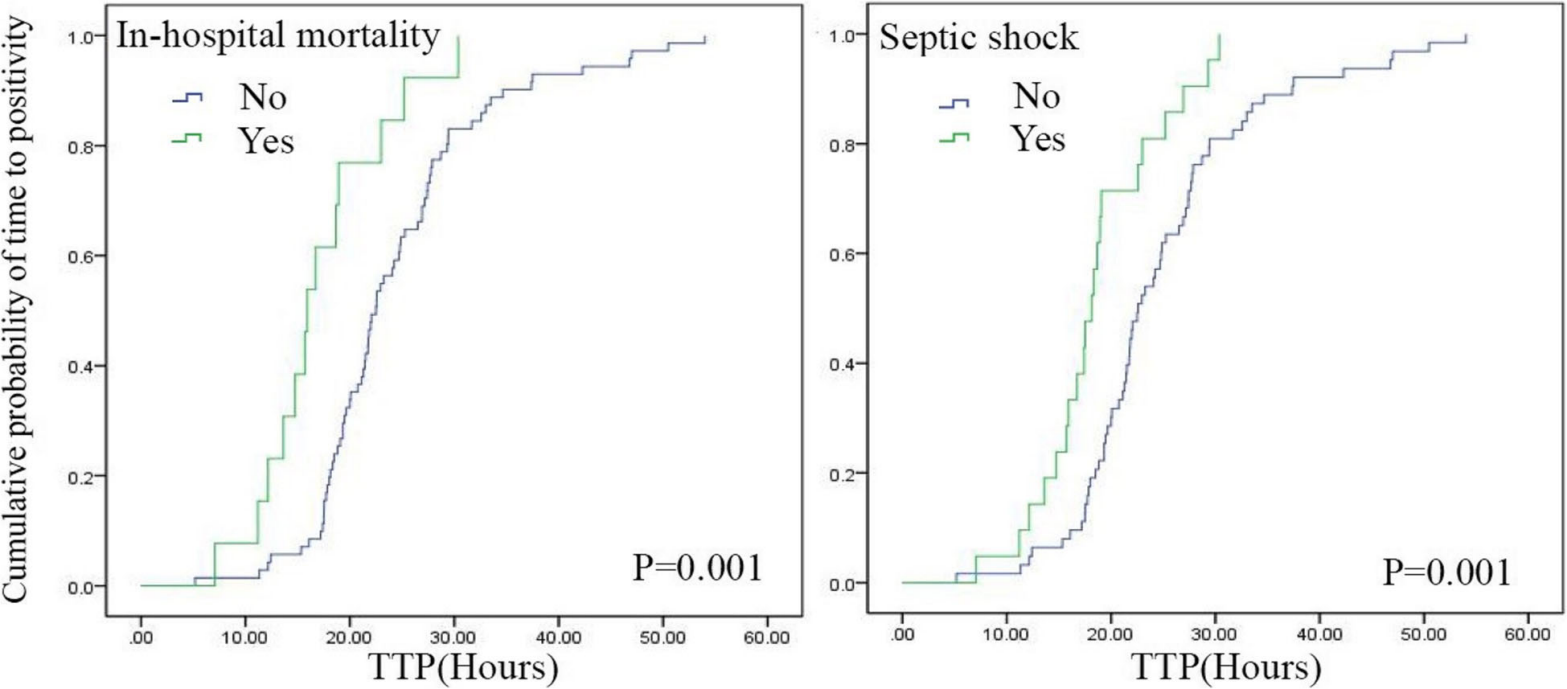
Comparison of TTP in initial blood culture series according to in-hospital mortality and septic shock. (A) Kaplan-Meier tests were plotted against in-hospital mortality. Cumulative probability of time to positivity of in-hospital mortality patients are presented as the green curve, while survival patients are shown in blue. The difference is significant (*P* = 0.001). (B) Kaplan-Meier tests plotted against septic shock. The green curve represents the cumulative probability of time to positivity of patients with septic shock, and the blue curve represents patients without, the difference being significant (*P* = 0.001)

**Fig. 4:**
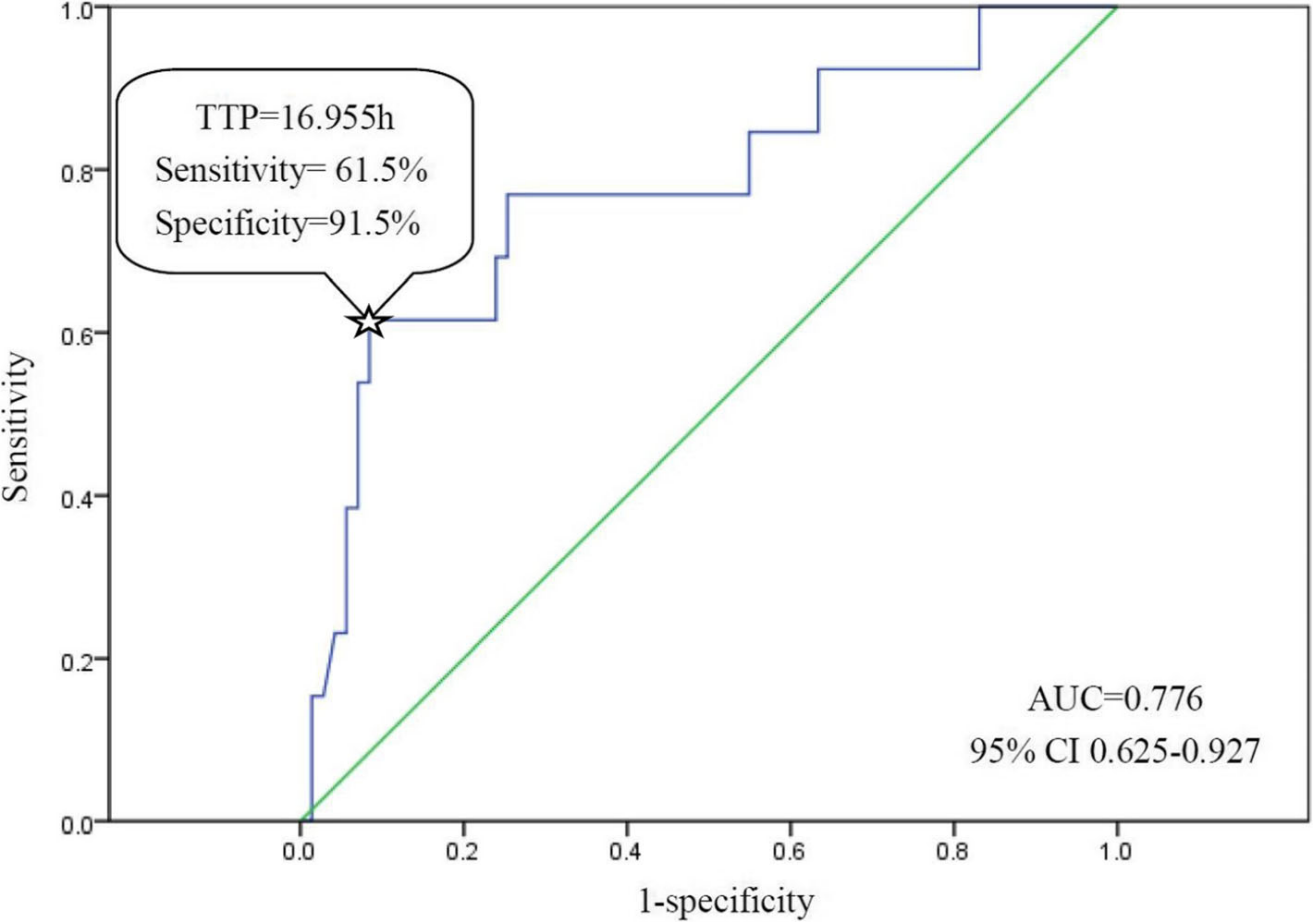
ROC curves of TTP to predict in-hospital mortality. The ROC curves were plotted according to the in-hospital mortality, and diagonal reference line is also shown. The X axis is 1-specificity, Y axis is sensitivity, and 16.955 h was the optimal cut-off point according to Youden’s index methodology. As shown above, ☆ points to the optimal cut-off, sensitivity is 61.5%, specificity is 91.5%, AUC (area under curve) is 0.776, with 95% CI 0.625–0.927

The median time to positivity (TTP) of blood culture of the 84 SAB children was 21.80 h (IQR 17.90, 27.42). The bar chart of TTP is shown in Fig. 2. The Kaplan-Meier tests of TTP analysis were constructed respectively with the categorical variables of outcomes. We observed a significant association of TTP with in-hospital mortality (*P* = 0.001) and septic shock incidence (*P* = 0.001) (Fig. 3). Receiver operating characteristic (ROC) curves of the TTP were plotted according to the primary outcome (in-hospital mortality) (See “Clinical outcomes” under Methods). Following Youden’s index methodology, we found 16.955 h to be the optimal cut-off point (61.5% sensitivity, 91.5% specificity, the area under the curves - AUC - were 0.776, with 95% CI 0.625–0.927) (Fig. 4). Therefore, we selected 17 h as the standard cut-off. Based on this, patients were divided into two groups: early detection group (TTP ≤17 h) and late detection group (TTP > 17 h).

### Clinical characteristics and outcomes comparison of early and late TTP group patients

A comparison between these two TTP groups was shown in Table 1. Demographic characteristics, the proportion of underlying conditions, APACHE II scores, hypoalbuminemia, prior antibiotic exposure, immunosuppressant use, nosocomial infection, methicillin-resistance and length of hospitalization – all were similar between the two groups. However, the other parameters showed important differences. In the early TTP group, 42.9% cases (6/14) needed invasive mechanical ventilation, but only 17.1% cases (12/70) in the late TTP group needed it (*P* = 0.033). Vasoactive agent was needed in 50.0% cases (7/14) in early TTP group but only in 18.6% cases (13/70) in the late TTP group (*P* = 0.012). In-hospital mortality was 57.1% (8/14) in early TTP group but 7.1% (5/70) in late TTP patients (*P* = 0.000). Septic shock occurred in 8 patients (8/14, 57.1%) in early TTP group and in 13 patients (13/70, 18.6%) in late TTP patients (*P* = 0.002). Taken together, these results indicated critical clinical differences between the early and late TTP groups.

**Table 1:** Clinical characteristics and outcomes comparison in early and late detection groups

Variables	Early Detection Group (*n* = 14)	Late Detection Group (*n* = 70)	P
Demographic characteristics			
Age, Median (IQR)	75.00 (16.25–143.50)	49.50 (8.49–124.75)	0.275
Weight, Median (IQR)	23.00 (8.50–36.25)	14.50 (7.50–32.75)	0.506
Male (n, %)	11 (78.6%)	44 (62.86%)	0.262
Underlying Conditions			
Hematologic malignancy (n, %)	2 (14.3%)	8 (11.43%)	0.765
Congenital heart disease (n, %)	4 (28.6%)	10 (14.29%)	0.193
Neutropenia (n, %)	2 (14.3%)	8 (11.43%)	0.765
Primary immunodeficiency (n, %)	2 (14.3%)	5 (7.14%)	0.394
Chronic kidney disease (n, %)	1 (7.1%)	2 (2.86%)	0.433
Source of infection			
Multiple sites^**b**^	5 (35.7%)	19 (27.1%)	0.519
Pneumonia	3 (21.4%)	20 (28.6%)	0.587
Bone or joint	0 (0.0%)	13 (18.6%)	0.081
Skin or soft tissue	0 (0.0%)	9 (12.9%)	0.158
Primary bacteremia	0 (0.0%)	4 (5.7%)	0.362
Intravenous catheter	2 (14.3%)	2 (2.9%)	0.068
Catheter related infection^**c**^	1 (7.1%)	1 (1.4%)	0.203
Incision	0 (0.0%)	2 (2.9%)	0.525
Endocarditis	2 (14.3%)	0 (0.0%)	**0.001** ^**a**^
CNS infection	1 (7.1%)	0 (0.0%)	**0.025** ^**a**^
APACHE II score ≥ 15 (n, %)	5 (35.7%)	22 (31.4%)	0.755
Prior antibiotic exposure (n, %)	7 (50.0%)	28 (40.0%)	0.491
Appropriate antibiotic exposure (n, %)	1 (7.1%)	12 (17.1%)	0.348
Immunosuppressants use (n, %)	6 (42.9%)	14 (20.0%)	0.068
Nosocomial infection (n, %)	3 (21.4%)	11 (15.7%)	0.603
Methicillin-resistance (n, %)	4 (28.6%)	25 (35.7%)	0.610
Hypoalbuminemia (n, %)	1 ((7.1%)	5 (7.1%)	1.000
Need for Treatments			
Invasive Mechanical ventilation (n, %)	6 (42.9%)	12 (17.1%)	**0.033** ^**a**^
Vasoactive agent (n, %)	7 (50.0%)	13 (18.6%)	**0.012** ^**a**^
Outcomes			
In-hospital mortality (n, %)	8 (57.1%)	5 (7.1%)	**0.000** ^**a**^
Septic shock (n, %)	8 (57.1%)	13 (18.6%)	**0.002** ^**a**^
Length of hospitalization Median (IQR)	17.00 (2.75–33.00)	22.50 (14.75–37.00)	0.151

^a^showed difference in the early and late detection groups (*P* < 0.05)

^b^8 cases were bone or joint + skin or soft tissue, 5 cases were bone or joint + skin or soft tissue + pneumonia, 4 cases were skin or soft tissue + pneumonia, 5 cases were bone or joint + pneumonia, 2 cases were endocarditis + pneumonia

^c^including urethral catheters and thoracic drain tube

### Risk factors of in-hospital mortality

Univariate logistic regression test demonstrated that in-hospital mortality was correlated with hematologic malignancy, immunosuppressants use, need for invasive mechanical ventilation and vasoactive agent, neutropenia, APACHE II score ≥ 15 and early TTP. Multivariable logistic regression test showed that early TTP, need for vasoactive agent were independent risk factors of in-hospital mortality (Table 2).

**Table 2:** Logistic regression analyses of in-hospital mortality

Variables	Univariate			Multivariate		
	OR	95% C.I	P	OR	95% C.I	P
Early TTP	17.333	4.293–69.984	0.000	97.494	4.477–2123.283	0.004
Need for vasoactive agent	34.444	6.475–183.236	0.000	40.513	2.988–549.355	0.005
APACHE II score ≥ 15	6.625	1.817–24.151	0.004	–	–	–
Neutropenia	4.815	1.136–20.413	0.033	–	–	–
Need for invasive mechanical ventilation	8.540	2.263–32.233	0.002	–	–	–
Immunosuppressants use	12.273	3.208–46.955	0.000	–	–	–
Hematologic malignancy	4.815	1.136–20.413	0.033	–	–	–

### Risk factors of septic shock

The univariate logistic regression test demonstrated that the incidence of septic shock was correlated with hematologic malignancy, immunosuppressants use, need for invasive mechanical ventilation and vasoactive agents, neutropenia, APACHE II score ≥ 15, and early TTP. As before, multivariable logistic regression showed that early TTP, need for vasoactive agent and APACHE II score ≥ 15 were independent risk factors of incidence of septic shock (Table 3).

**Table 3:** Logistic regression analyses of septic shock incidence

Variables	Univariate			Multivariate		
	OR	95% C.I	P	OR	95% C.I	P
Early TTP	5.846	1.730–19.761	0.004	6.757	1.094–41.730	0.040
Need for vasoactive agent	44.250	10.571–185.228	0.000	27.296	5.459–136.488	0.000
APACHE II score ≥ 15	10.625	3.410–33.108	0.000	9.821	1.885–51.174	0.007
Need for invasive mechanical ventilation	11.611	3.435–39.253	0.000	–	–	–
Neutropenia	5.900	1.475–23.600	0.012	–	–	–
Immunosuppressants use	9.167	2.935–28.629	0.000	–	–	–
Hematologic malignancy	5.900	1.475–23.600	0.012	–	–	–

## Discussion

The prognostic role of TTP in SAB children remained unclear, which prompted us to conduct the present study. We explored the correlation between TTP and clinical characteristics and clinical outcomes in SAB children. As noted above, the median TTP in this group of children was 21.80 h (IQR 17.90, 27.42), and the optimal TTP cut-off was 16.955 h, which were longer than those of adult SAB patients having the optimal TTP cut-off of 12 or 14 h [10, 11, 16]. TTP of blood culture could be influenced by many factors, such as the inoculum, the volume of blood drawn, the incubation conditions and the presence of growth [21]. Although the incubation conditions were stable in our study, only 3 ml blood was extracted to inoculate from our pediatric patients, which was much less than 10 ml in adults. Thus, despite the higher bacterial load in children, this relatively less blood volumes may lead to the long TTPs. Kim et al. [16] stated that the TTP of MRSA was longer than that of MSSA, and nearly one thirds of the bacteria in our study were MRSA, which could also contribute to long TTPs. Our results inferred that adult and childhood SAB patients may present different TTPs; this is consistent with the previous study by Hal SJV et al. [12], which found that age was a predictor of mortality.

To our knowledge, this is the first study to explore the relationship between TTP and clinical outcomes of SAB in children. Here, we have documented that early TTP group patients had 97-fold higher in-hospital mortality and 7-fold higher incidence of septic shock than those with late TTPs. Early TTP was an independent risk factor of in-hospital mortality and septic shock incidence, which indicated that early TTP is indeed associated with worse clinical outcomes in children with SAB. The concept of early TTP of blood cultures is directly related with a higher bacterial load in blood. This is in line with the previous studies in adults, in which early TTP was considered an indicator of poor clinical outcomes [10, 16, 17, 22, 23].

We also documented that patients needing vasoactive agents had a 40-fold higher in-hospital mortality and 27-fold higher incidence of septic shock. Need for vasoactive agent was in fact another independent risk factor of in-hospital mortality and septic shock. Sepsis has been described as a paradigm of acute whole body inflammation with systemic damage in the vascular endothelium [24], and it is well documented that sepsis patients suffered from endothelial disruption and damage [25]. Such damages may result in an impairment of tissue and whole body respiration despite adequate O_2_ supply. This may also indicate an association with severity and mortality. Not surprisingly, as Badia et al. [26] showed that the need for vasoactive agent is associated with increased risk of mortality in bacteremia patients.

In addition, patients with APACHE II scores≥15 also presented 10-fold incidence of septic shock, and a statistically significant difference in early TTP group. APACHE II score has been advocated as the gold standard for risk evaluation in critically ill patients [27], and could partly reflect the patient’s underlying conditions, translating into unfavorable outcomes [28]. Although APACHE II score has not been used routinely in children these years, some studies demonstrated it can serve as a prognosis factor of children with AKI [29], ARDS [30] and peritonitis [31] effectively. Meanwhile, there were also trends for correlation with hematologic malignancy and neutropenia and immunosuppressant use. These underlying conditions may affect the TTPs and outcomes, consistent with the previous studies [1, 17, 22]. Immune status is also one of the determinants of bacteremia severity [17, 32, 33], and therefore, plays a crucial role in bacteremia patients [22]. Immunosuppressants, widely used in patients with hematologic malignancy, may in fact inhibit the activity of immunocytes [34, 35], leading to neutropenia and decreased bacteria clearance, finally resulting in higher bacterial loads and severe bacteremia.

Our finding that prior antibiotic exposure and appropriate antibiotic exposure had little impact on TTPs and clinical outcomes is consistent with other studies [16, 17, 22], while differing from those on adults [36, 37]. Nevertheless, van Paridon et al. [38] stated that the timing of antibiotic use in children, presented early use of appropriate antibiotics, had little association with sepsis outcomes. As the details of administration were hardly found in this retrospective sudy, further prospective investigations are needed to determine the effects of appropriate antibiotic exposure on TTPs and clinical outcomes.

Lastly, we would like to address the potential limitations of this study. First, as a single- center study, the data were collected from one academic teaching hospital in China, the results may not easily extrapolate to patients admitted to other hospitals or in other countries. Second, the relatively small sample size of our study (*n* = 84) may result in type II error and reduce the ability to determine the statistical significance of the variables. Third, as the data were collected from the medical records retrospectively, some information was unfortunately missed. Clearly, multi-center, prospective studies in the future, using a larger sample size, should strengthen the results of this study.

## Conclusion

Early TTP, need for vasoactive agent were independent risk factors of in-hospital mortality; early TTP, need for vasoactive agent and APACHE II score ≥ 15 were independent risk factors of septic shock in *S. aureus* bacteremia children. TTP of ≤17 h may correlate with the worse outcomes for these children.

## Abbreviations

ANC: Absolute neutrophil count
APACHE II scores: acute physiology and chronic health evaluation II scores
CI: Confidence interval
CNS: Central nervous system
IQR: Interquartile range
MRSA: Methicillin-resistant *Staphylococcus aureus*
MSSA: Methicillin-Sensitive *Staphylococcus aureus*
OR: Odds ratio
ROC: Receiver operating characteristic
SAB: *Staphylococcus aureus* bacteremia
SIRS: Systemic inflammation reaction syndrome status
TTP: Time to positivity
