# Correction to: Time to positivity of blood culture is a risk factor for clinical outcomes in *Staphylococcus aureus* bacteremia children: a retrospective study

**DOI:** 10.1186/s12879-019-4194-x

**Published:** 2019-07-11

**Authors:** Yuanyuan Li, Qinyuan Li, Guangli Zhang, Huan Ma, Yi Wu, Qian Yi, Lili Jiang, Jiao Wan, Fengtao Suo, Zhengxiu Luo

**Affiliations:** 1 Key Laboratory of Pediatrics in Chongqing, Chongqing, China; 2 Department of Children’s Hospital of Chongqing Medical University of Education, Ministry of Education Key Laboratory of Child Development and Disorders, China International Science and Technology Cooperation base of Child development and Critical Disorders, Chongqing, China; 3 Department of Respiratory Medicine, Children’s Hospital of Chongqing Medical University, Chongqing, 401122 China

**Correction to: BMC Infectious Diseases (2019) 19:437.**

**https://doi.org/10.1186/s12879-019-3993-4**

Following publication of the original article [1], the author reported two errors in Fig. 1 and Fig. 2.

**Fig. 1:**
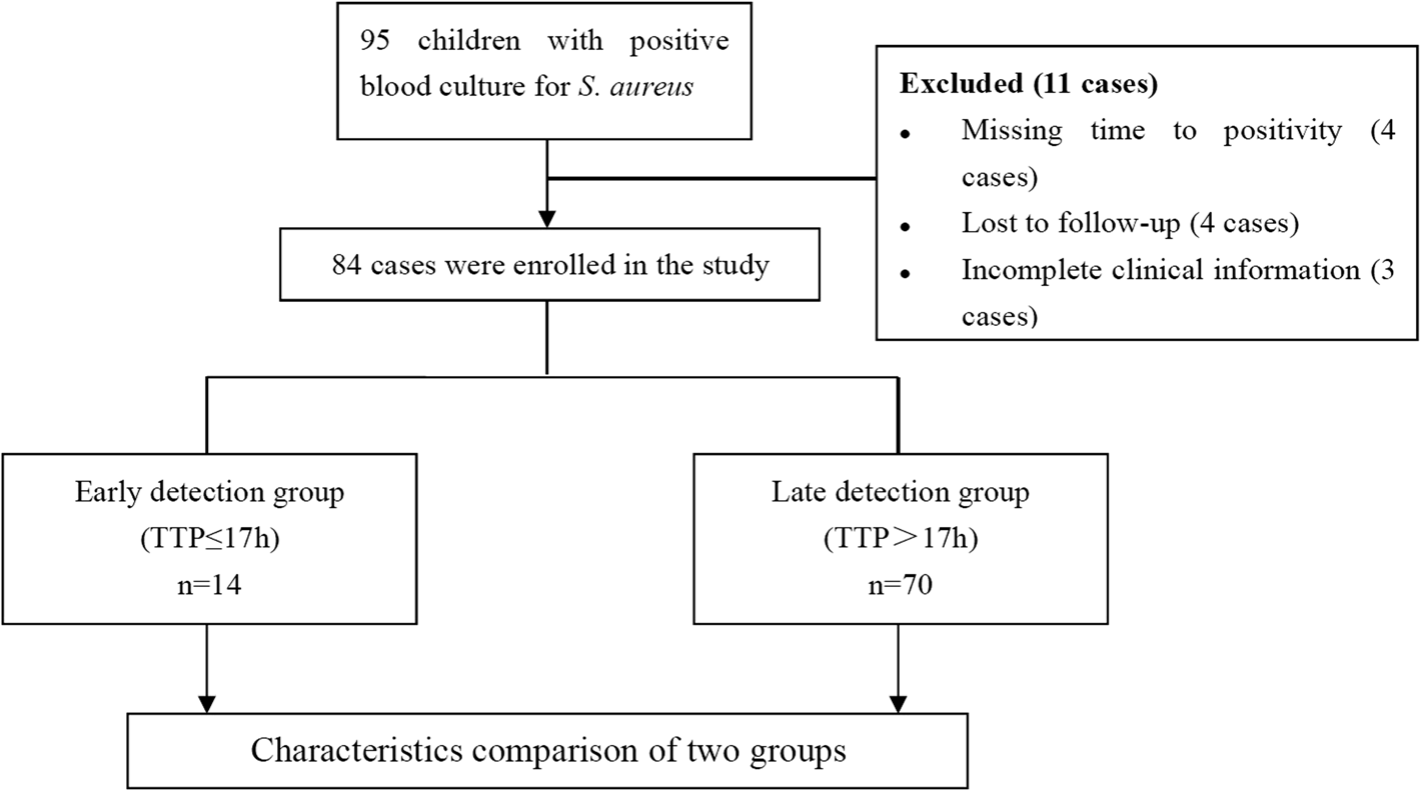
Flow diagram of the population. As shown, a total of 84 cases were enrolled according to the inclusion and exclusion criteria, and then divided into early detection group (TTP ≤ 17 h) and late detection group (TTP > 17 h). The clinical characteristics of each group were then determined

**Fig. 2:**
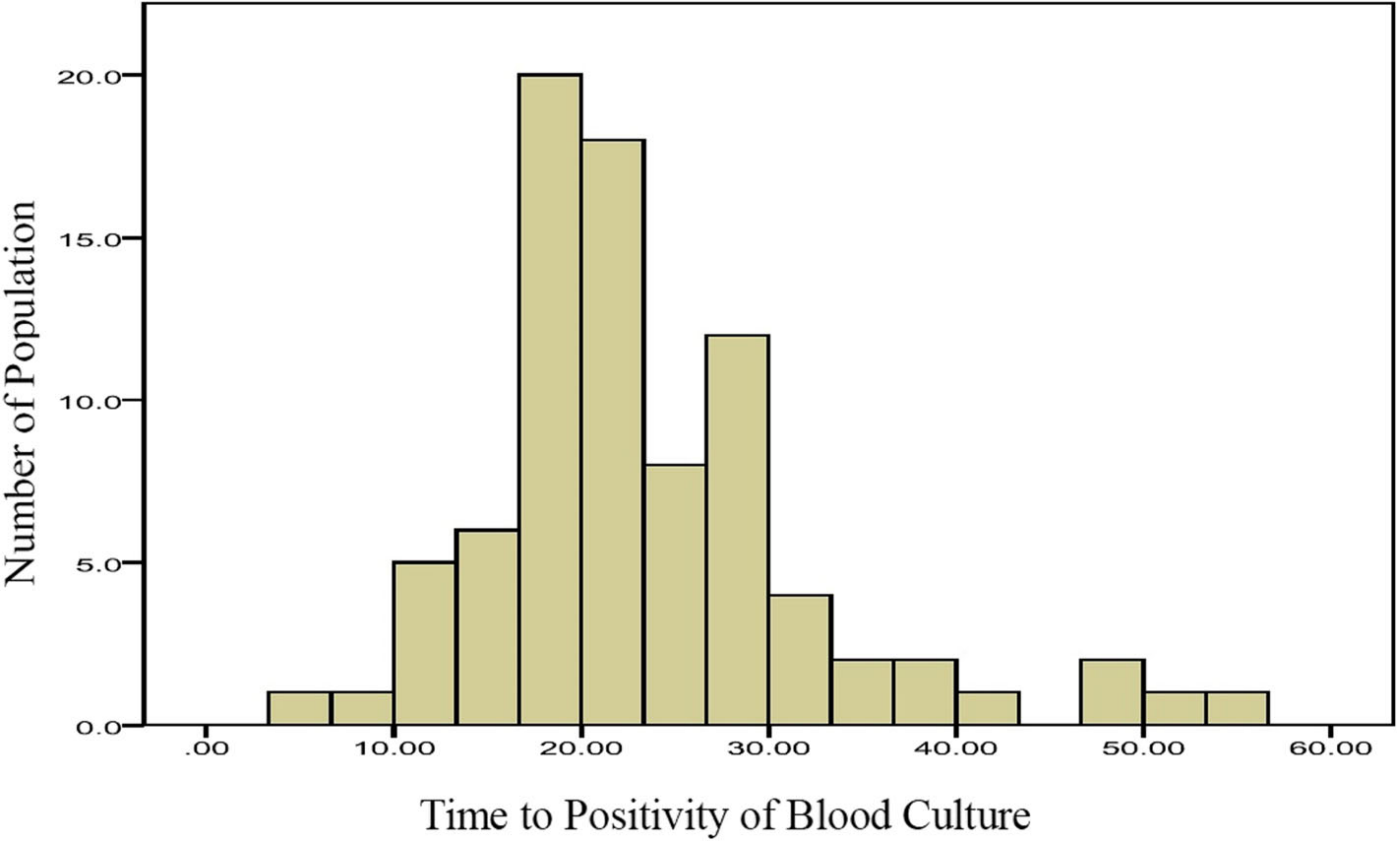
Bar chart of TTP in *Staphylococcus aureus* bacteremia children. Out of a total of 84 SAB children, the number of children in each TTP period was plotted against the corresponding TTP, as shown. Details of the quantification have been described in Methods

The correct figures can be found below.

The corrections have been implemented in the original article as well.

The publisher apologized for any inconvenience this might has caused.
